# Supplementary material for: Mesoscopic structural phase progression in photo-excited VO2 revealed by time-resolved x-ray diffraction microscopy
Source: Sci Rep. 2016 Feb 26;6:21999. doi: 10.1038/srep21999 (PMC4768076; doi:10.1038/srep21999)
Supplement: Supplementary Information [file srep21999-s1.pdf]

Supplemental Materials for "Mesoscopic structural phase progression in photo-excited VO_2 revealed by time-resolved x-ray diffraction microscopy"

Yi Zhu[1], Zhonghou Cai[1], Pice Chen[2, †], Qingteng Zhang[2, §], Matthew J. Highland[3], Il Woong Jung[4], Donald A. Walko[1], Eric M. Dufresne[1], Jaewoo Jeong[5], Mahesh G. Samant[5], Stuart S. P. Parkin[5,6], John W. Freeland[1], Paul G. Evans[2], Haidan Wen[1]*

[1] Advanced Photon Source, Argonne National Laboratory, Argonne, Illinois 60439, USA
[2] Department of Materials Science and Engineering, University of Wisconsin–Madison, Madison, Wisconsin 53706, USA
[3] Materials Science Division, Argonne National Laboratory, Argonne, Illinois 60439, USA
[4] Center for Nanoscale Materials, Argonne National Laboratory, Argonne, Illinois 60439, USA
[5] IBM Almaden Research Center, San Jose, California 95120, USA
[6] Max Planck Institute for Microstructure Physics, Halle 06120, Germany

*Corresponding author: wen@aps.anl.gov
[†] Present address: Department of Materials Science and Engineering, Northwestern University, Evanston, Illinois 60208, USA
[§] Present address: Advanced Photon Source, Argonne National Laboratory, Lemont, Illinois 60439, USA

1. Technical details of the experimental setup

The laser pump, hard x-ray microdiffraction imaging probe experiment was carried out at the 7-ID-C beamline of the Advanced Photon Source (APS) (Fig. S1). An ultrafast Ti:Sapphire laser system (Micra and Legend from Coherent Inc.) provides laser pulses with 60 fs pulse duration and 1.55 eV photon energy at 1 kHz rate to photo-excite a thin film VO_2 sample grown on Al_2O_3 (001) substrate. The laser pulse is focused to ~ 200 μm (FWHM) in diameter on the sample surface.

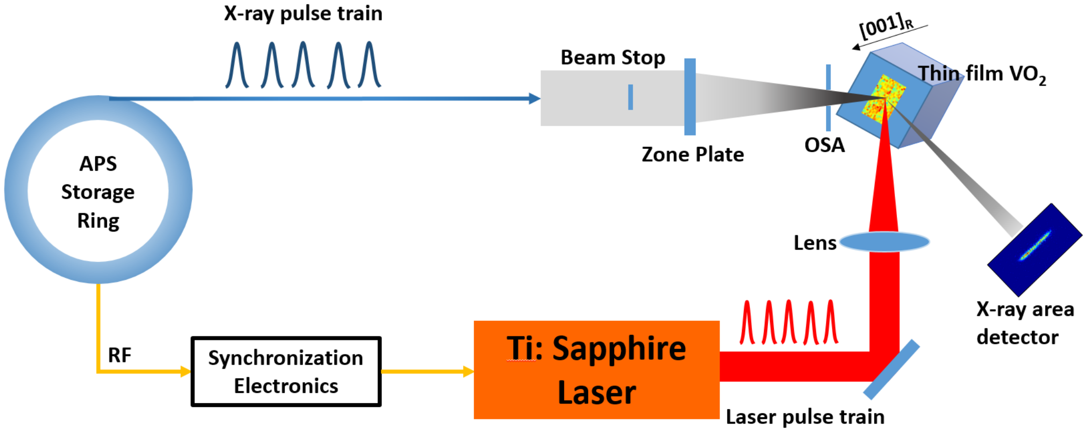

Figure S1 Experimental setup. A femtosecond laser pulse train is synchronized to an x-ray pulse train from the Advanced Photon Source (APS) storage ring. The x-ray pulses are focused at the sample position by a zone plate with a diameter of 160 μm and an out-zone width of 100 nm. The femtosecond laser pulses are focused onto the sample surface and overlapped with the x-ray probe beam. The x-rays horizontally diffracted from VO_2 are detected by an x-ray area detector.

Monochromatic 10 keV hard x-ray pulses with 0.1% band width from the APS synchrotron storage ring are focused by an x-ray Fresnel zone plate. The beam stop and the optical sorting

aperture (OSA) are employed to block the central non-diffracted x-ray component and the higher order diffracted x-ray component respectively. The incident x-ray flux through the zone plate with a diameter of 160 μm is estimated to be ~4×10^{10} photons per second. At the sample position, the flux is reduced to 3.2×10^{9} photons per second, in agreement with the 8% efficiency of the zone plate. The x-ray footprint on the sample is 350 nm and 830 nm (FWHM) in the vertical and horizontal directions respectively. Spatial overlap of the pump and probe beams is achieved by steering the laser beam to be concentered with the x-ray beam on the sample, aided by a micrometer-sized phosphor particle that fluoresces under the illumination of both optical and x-ray beam.

The ultrafast laser system is electronically synchronized to the RF (radio frequency) signal of the APS storage ring with a timing jitter less than 10 ps. The arrival time of laser pulses to the sample can be controlled electronically with respect to that of the x-ray pulses using a delay generator. The time resolution of the experiment is determined by the probe x-ray pulse duration of 100 ps.

The thin film VO_2 sample is mounted on a XYZ nano-positioning piezo stage (nPoint, Inc.) with the *c*-axis along the out-of-plane direction. The piezo stage is mounted to the inner circle of a two-circle diffractometer. During the experiment, a 10×10 μm^2 region is raster scanned using the piezo stage with a step size of 250 nm. This field of view is chosen so that the area is small enough to be homogenously excited by an optical pulse and big enough to illustrate the phase transition spatial features.

The x-ray diffraction peaks from the monoclinic (M) and rutile (R) phases are measured with an x-ray area detector (Pilatus 100K, DECTRIS Ltd.) mounted on the outer circle of the diffractometer. The repetition rate of the x-ray pulses is 6.5 MHz. Only the x-ray pulses that are paired with the excitation laser pulses are recorded by gating the detector at 1 kHz laser repetition rate, providing an effective time-resolved x-ray flux of ~10^{6} photons per second at the sample position.

2. One-dimensional thermal transport

The out-of-plane phase progression as discussed in the main text shown in Fig. 2(c) is driven by thermal transport. This is due to inhomogeneous optical excitation along the out-of-plane direction as the film thickness is twice as thick as the penetration depth of the 800 nm excitation laser pulse. As a result, only surface layers of the VO_2 film can be sufficiently excited to the R phase at low excitation fluence, while the x-ray pulse is able to probe the film thoroughly in depth. The progression of this phase boundary deeper into the film results in the increase of R phase composition in the probed region following the excitation. To quantitatively study the dynamics associated with the out-of-plane phase progression, we use the intensity change of the Bragg diffraction peak of the M phase to gauge the fractional composition of the excited VO_2 along the depth of the probed region. The diffraction intensity at the fixed angle of 25.62 degree is corrected as the integrated intensity change of the Bragg peak based on the temperature-dependent XRD measurements. Using this intensity-to-thickness calibration, at 100 ps (t_1) after optical excitation with a pump laser fluence of 14 mJ/cm^2 (shown by the purple curve), we find that the M phase decreased by 14%, corresponding to 28 nm-thick VO_2 that is transformed into the R phase. The thermalization of the film leads to a reduction of thermal gradient at the phase boundary. The progression eventually loses the driving force (thermal gradient) for the out-of-plane direction at t_2, which is defined as the time when the fast intensity reduction transits into a slower linear reduction, shown as the dashed horizontal lines in Fig. S2. As the diffraction intensity of the M phase further decreases by 36% at t_2, the thickness of R phase increases from 28 nm to 73 nm (Fig. S2). At a higher pump fluence of 24 mJ/cm^2, thicker layer of the film is converted from M to R phase after the optical excitation, which results in a larger initial diffraction intensity change at 100 ps. The out-of-plane progression ends at later time t_3 with a phase boundary progression from 86 to 132 nm. The time scales of

the out-of-plane phase progression agree with a thermal diffusion process. At both pump fluences, the phase progression over a length of $x \approx 50$ nm is about 1 ns. This is consistent with the thermal diffusion time of $t \approx \frac{x^2}{D} = 1.25$ ns, where D=0.02 cm^2/s is the thermal diffusion coefficient of VO_2 [S1].

The magnitude of the initial change of the diffraction intensity at 100 ps is proportional to the density of the nucleation sites that can transform upon optical excitation. At lower pump fluence, fewer sites in the illuminated volume of VO_2 will be converted directly by absorption of photons and yield smaller number of pinning sites, as seen by the smaller change at t_1 in the pink and blue curves comparing to the red and black curves in Fig. S2. As the pump fluence increases, the number of sites that can be excited across the phase transition also increases, leading to larger magnitude of the intensity change.

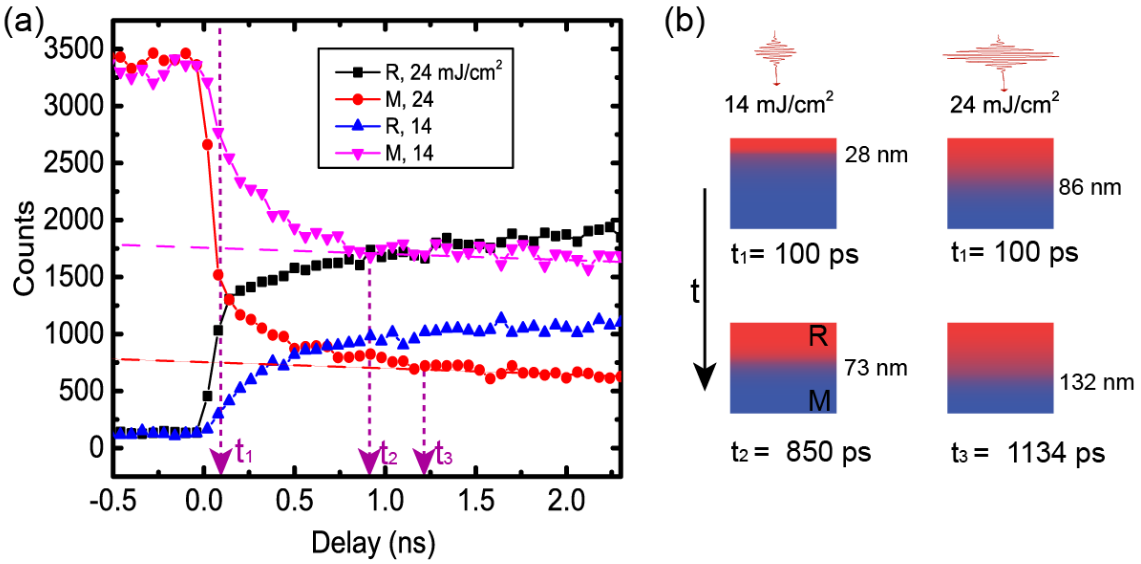

Figure S2 (a) Diffraction intensity of M and R phases as a function of time excited at two optical incident fluences. The purple dashed arrows show the start (t_1=100 ps, limited by x-ray pulse duration) and end (t_2, t_3) time of the longitudinal phase progression. The dashed horizontal lines are linear fits for the slow components on several ns time scales. (b) The schematics of the out-of-plane phase progression at two pump fluences. The red and blue regions indicate the layers of R and M lattice.

The change of diffraction intensity within ~1 ns can be fitted by a one-dimensional thermal transport model (Fig. S3). The thermal transport simulation was based on the VO_2/Al_2O_3 heterostructure using similar approach in Ref. [S2]. The initial temperature profile of VO_2 film is exponentially dependent on the position z along the depth direction:

$$\mathrm{T}(0 < z < D, t = 0) = \mathrm{T}_0 \exp(-\alpha z),$$

where α is the absorption coefficient of 800 nm light. The temperature profile as a function of time t was obtained by numerically solving the one-dimensional heat equation. As heat flows from the surface toward deeper VO_2 layers, larger volume of VO_2 is heated above the transition temperature, driving the progression of the R-M phase boundary into deep layers. The R-M phase boundary is defined by the layer of VO_2 at the transition temperature of 338 K. We track the position of R-M phase boundary and plot its change as a function of time in Fig. S3. It is in agreement with the measurements of the R phase diffraction intensity change.

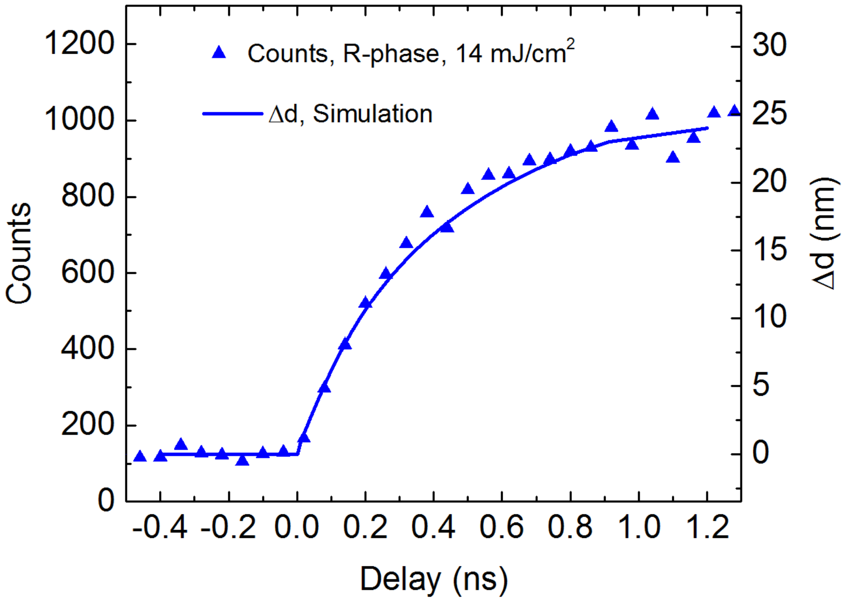

Figure S3 The diffraction intensity of R phase as a function of time is compared with the simulation result. The solid curve shows the change of R phase thickness as a function of time, driven by thermal transport along the out-of-plane direction.

References:

[S1] V. A. Lobastov, J. Weissenrieder, J. Tang, and A. H. Zewail, Nano Lett. **7**, 2552 (2007).

[S2] H. Wen, L. Guo, E. Barnes, J. H. Lee, D. A. Walko, R. D. Schaller, J. A. Moyer, R. Misra, Y. Li, E. M. Dufresne, D. G. Schlom, V. Gopalan, and J. W. Freeland, Phys. Rev. B **88**, 165424 (2013).
